# Uncovering the biogeography of the microbial commmunity and its association with nutrient metabolism in the intestinal tract using a pig model

**DOI:** 10.3389/fnut.2022.1003763

**Published:** 2022-09-26

**Authors:** Yuanyuan Song, Kai Chen, Lu Lv, Yun Xiang, Xizhong Du, Xiaojun Zhang, Guangmin Zhao, Yingping Xiao

**Affiliations:** ^1^State Key Laboratory for Managing Biotic and Chemical Threats to the Quality and Safety of Agro-products, Institute of Agro-product Safety and Nutrition, Zhejiang Academy of Agricultural Sciences, Hangzhou, China; ^2^Key Laboratory of Vector Biology and Pathogen Control of Zhejiang Province, School of Life Sciences, Huzhou University, Huzhou, China; ^3^Quality and Safety of Animal Products Group, Zhejiang Center of Animal Disease Control, Hangzhou, China; ^4^Institute of Animal Husbandry and Veterinary Medicine, Jinhua Academy of Agricultural Sciences, Jinhua, China

**Keywords:** gut microbiota, short-chain fatty acids, nutrients, amino acid, intestinal tract

## Abstract

The gut microbiota is a complex ecosystem that is essential for the metabolism, immunity and health of the host. The gut microbiota also plays a critical role in nutrient absorption and metabolism, and nutrients can influence the growth and composition of the gut microbiota. To gain a better understanding of the relationship between the gut microbial composition and nutrient metabolism, we used a pig model by collecting the contents of the different intestinal locations from six pigs to investigate microbial composition in different intestinal locations based on 16S rRNA gene sequencing and the concentrations of short-chain fatty acids (SCFAs), amino acids, fat, and crude ash in different intestinal locations using gas chromatography and chemical analysis. The results showed that the richness and diversity of intestinal microbial communities gradually increased from the small intestine to the large intestine. The relative abundance of Proteobacteria was higher in the jejunum and ileum, whereas the proportion of Firmicutes was higher in the cecum and colon. The concentrations of SCFAs were higher in the cecum and colon (*P* < 0.05). The concentrations of amino acids were higher in the small intestine than in the large intestine, while the amino acid content was significantly higher in the ascending colon than in the transverse colon and descending colon. The correlation analysis revealed that *Ruminococcaceae UCG-005, Coriobacteriaceae_uncultured, [Eubacterium] hallii* group, *Mogibacterium* and *Lachnospiraceae AC2044* group had a higher positive correlation with SCFAs, crude ash and fat but had a negative correlation with amino acids in different gut locations of pigs. These findings may serve as fundamental data for using nutrient metabolism to regulate human and animal gut microbes and health and provide guidance for exploring host-microbe bidirectional interaction mechanisms and driving pathways.

## Introduction

The gastrointestinal tract (GIT) is the largest interface between the external and internal environments and contains the largest amount and the greatest diversity of microorganisms. Humans and mammals are colonized with a vast and complex microbial community, which is known to play an important role in host metabolism, including digestion and absorption of nutrients, energy acquisition, carbohydrate metabolism and the immune system (1–4). Along the longitudinal axis of the intestinal lumen, distinct microhabitats selectively harbor characteristic microbes (5, 6).

The gut microbiota can directly affect the utilization and absorption of nutrients, including carbohydrates, lipids, proteins and minerals, and are also regulated by the host nutrition (6–9). SCFAs are produced by gut saccharolytic microbes through fermenting and degrading indigestible carbohydrate (10, 11), which can provide energy for intestinal epithelial cells and serve as an energy source for the growth of anaerobic bacteria (12, 13). *Lachnospiraceae_uncultured* may be a potential beneficial bacterium involved in the metabolism of a variety of carbohydrates, and the fermentation product acetic acid is the main source of bacteria that provides energy for the host. The fermentation metabolites of *Ruminococcaceae* (*CG-004, 05, 013, 014*) are mainly acetic acid and formic acid, and bacteria in the Prevotella-dominated enterotype mainly absorb monosaccharides and degrade mucins to obtain energy (14, 15). Some species of the genus *[Eubacterium] rectale* group can ferment carbohydrates in food and produce large amounts of acetic acid and butyric acid (16). In addition, acetate produced by *Bacteroides, Bifidobacterium, Prausnitzii*, and *Ruminococcus* acts on the brain through the blood–brain barrier (17). The gut microbiota may play important roles in the host amino acid balance and health through multiple pathways (18, 19). Bacteria have various metabolic pathways for amino acid metabolism, such as the synthesis of cellular proteins and amino acid catabolism (20). Studies have shown that small intestinal bacteria may mainly utilize amino acids for the synthesis of bacterial proteins, while large intestinal bacteria mainly use them for catabolism (21). Bacteria in the rumen and large intestine of animals and humans can degrade amino acids in large quantities. *Clostridium* can degrade amino acids through the Stickland reaction, and the main metabolites are branched-chain fatty acids and ammonia (22). Some studies have shown that amino acid metabolism by intestinal bacteria is ultimately performed for their survival and growth in the complex intestinal environment. For example, Lactobacillus can synthesize bacterial proteins by utilizing exogenous amino acids, which have an important role in host nutrition and physiology (23). The gut microbiota also synthesizes many essential amino acids, such as lysine, threonine, and arginine. For example, some intestinal microorganisms are able to bind NH_3_ from the breakdown of amino acids and resynthesize essential amino acids or proteins (24).

In recent years, numerous studies have shown a correlation between the gut microbiota and host metabolism (25–30), but the impact of the gut microbiota on nutrient metabolism remains unclear, and few studies have examined the metabolic compartmentalization of nutrients in different locations of the intestinal tract. Pigs are very similar to humans in terms of gastrointestinal tract development, physiology, digestive function and composition, the gut microbiota of pigs is 96% similar to humans in functional pathway (31–34). The minimum nutritional requirement of pigs is equivalent to the daily nutritional requirement of humans and the common physiological structure leads to a similar digestive tract transit time and nutrient absorption process, the pigs also can collect repeated measurement data (35). So pigs are an ideal model for studying human gut microbiota and nutrient metabolism. Herein, we investigated the intestinal microbiota structure in different parts of pigs and analyzed its association with the concentrations of SCFAs, amino acids, fat, and crude ash in different intestinal locations. The present findings could provide insights into the association between the microbial community and nutrient metabolism with gut homeostasis and whole-body health in humans and mammals.

## Materials and methods

### Ethics statement

All animal experiments were approved by the Institutional Animal Care of the Zhejiang Academy of Agricultural Sciences in accordance with the relevant rules and regulations (ZAAS-2017-009).

### Animal experiments and sample collection

Six female Jinhua pigs were randomly selected from the pig farm of Jinhua Academy of Agricultural Sciences, reared in the same environment and fed the same diet daily and water *ad libitum*. The detailed ingredients and nutrient contents of the diet are presented in Table 1. The pigs (71.90 ± 0.82 kg) were killed at the age of 250 days under anesthesia, and then the contents of the jejunum, ileum, cecum, ascending colon, transverse colon, and intermediate descending colon were collected immediately. We collected samples in sterile tubes and quickly frozen them in liquid nitrogen, and then transferred to a refrigerator at−80 °C for future chemical and microbial analyses.

**Table 1:** Composition and nutrient levels of basal diet.

**Items**	**Content**		
	**D 45~90**	**D 91~150**	**D 151~250**
**Ingredient** (%)
Corn	63.30	63.00	60.94
Soybean meal	28.44	20.99	20.50
Wheat bran	3.90	11.75	13.50
CaHPO_4_	0.30	1.20	1.20
Limestone	2.00	1.50	1.10
NaCl	0.26	0.26	0.26
Zeolite powder	0.50	1.40	1.40
*L*-Lysine HCL	0.30	0.20	0.10
Premix^a^	1.00	1.00	1.00
Total	100	100	100
**Nutrient level**^b^(%)
Digestible energy/(MJ·kg^−1^)	13.59	13.22	13.21
Crude protein	19.38	16.74	15.15
Calcium	0.87	0.85	0.85
Phosphorus	0.69	0.61	0.58
Lysine	1.12	0.80	0.96
Methionine + Cystine	0.56	0.54	0.57
Threonine	0.75	0.64	0.56
Tryptophan	0.23	0.20	0.18

^a^45 ~ 90 d The premix included (per kg of the diet): Cu 4.40 mg, Fe 70 mg, Mn 2.44 mg, Zn 67.10 mg, I 0.15 mg, Se 0.3 mg, VA 1 500 IU, VD_3_ 171.6 IU, VE 10 IU, VK_3_ 0.8 mg, VB_l_ 5 mg, VB_2_ 3.6 mg, VB_6_ 1.5 mg, VB_l2_ 10 mg, pantothenic acid 10 mg, nicotinic acid 30 mg, folic acid 300 mg, biotin 50 mg; 91 ~ 150 d: Cu 3.80 mg, Fe 55 mg, Mn 2.14 mg, Zn 58.10 mg, I 0.14 mg, Se 0.23 mg, VA 1 300 IU, VD_3_ 158.6 IU, VE 8 IU, VK_3_ 0.8 mg, VB_l_ 5 mg, VB_2_ 3.6 mg, VB_6_ 1.5 mg, VB_l2_ 10 mg, pantothenic acid 7.49 mg, nicotinic acid 20 mg, folica cid 280 mg, biotin 50 mg; 151 ~ 250 d:Cu 3.40 mg, Fe 9940 mg, Mn 1.44 mg, Zn 55.10 mg, I 0.14 mg, Se 0.17 mg, VA 1 286 IU, VD_3_ 149.5 IU, VE 7 IU, VK_3_ 0.8 mg, VB_l_ 5 mg, VB_2_ 3.6 mg, VB_6_ 1.5 mg, VB_l2_ 10 mg, folic acid 260 mg, pantothenic acid 7.11 mg, nicotinic acid 10 mg, biotin 50 mg.

^b^DE, CP, Ca, TP, Lys and Met + Cys were calculated values.

### DNA extraction, sequencing, and data analysis

The CTAB/SDS method was used to extract genomic DNA from 36 samples. DNA concentration and purity were monitored on a 1% agarose gel and DNA was diluted to 1 ng/μl using sterile water according to the concentration. The 16S rRNA gene V4-V5 region was amplified using primers (515F: 5′- GTGCCAGCMGCCGCGGTAA-3′; 907R: 5′-CCGTCAATTCCTTTGAGTTT-3′) with barcodes. All PCRs were conducted in 30 μL reactions with 15 μL of Phusion^®^High-Fidelity PCR Master Mix (New England Biolabs), 0.2 μM forward and reverse primers, and approximately 10 ng of template DNA. Initially, the samples were denaturated at 98 °C for 1 min, followed by 30 cycles of denaturation at 98 °C for 10 s, annealing at 50 °C for 30 s, elongation at 72 °C for 60 s and finally 72 °C for 5 min. We used A GeneJET Gel Extraction Kit (Thermo Scientific) to purify the PCR mixture and NEB Next^®^Ultra^TM^ DNA Library Prep Kit for Illumina (NEB, Ipswich, MA, USA) to prepare sequencing libraries following the manufacturer's recommendations, and index codes were added. The Illumina paired-end reads were filtered and demultiplexed (36), merged into tags using FLASH, and assorted to each sample according to the attached barcode (37).

The RDP classifier was used to performs taxonomy assignment of the OTUs (38). We calculated and visualized the alpha diversity (Chao 1 estimator, Shannon, and Simpson indices) using GraphPad Prism 8 (GraphPad software, San Diego, CA, USA). The differences in microbial communities in different intestinal segments using principal coordinate analysis (PCoA). An analysis of linear discriminant analysis (LDA) coupled with effect size (LEfSe) were carried out to identify differentially abundant features between groups (39).

### SCFA measurement

As described in our previous study, the concentrations of SCFAs in intestinal contents were detected by gas chromatography (40, 41). Briefly, 0.1 g intestinal contents of each bowel segment were weighed into a 1.5–mL centrifuge tube and suspended in Milli-Q water (9 volumes). After centrifugation (12 000 rpm, 10 min), the supernatant (0.5 mL) was collected and mixed with 0.1 mL of 25% (w/v) metaphosphoric acid and crotonic acid solution and stored at −20 °C overnight. The samples were filtered with a microporous membrane (0.22 μm), and the continuous filtrate was collected for analysis. The levels of SCFAs in each bowel content were determined using gas chromatography (GC) (Shimadzu, Kyoto, Japan), and the chromatographic column was a capillary column (InertCap FAPF). The chromatographic conditions were as follows: FID detector operating at 180 °C, column at 110 °C, vaporization chamber at 180 °C, and carrier gas nitrogen at 0.06 MPa, while the auxiliary gas consisted of hydrogen and air at pressure of 0.05 MPa and 0.05 MPa, respectively.

### Chemical analysis

Samples of intestinal contents were freeze-dried. The procedure set by the Association of Official Analytical Chemists (AOAC, 2000) was used to determine the concentrations of moisture, crude fat and crude ash. We used a Hitachi L-8900 amino acid analyzer (Hitachi, Tokyo, Japan) to determine the contents of all the amino acids following acid hydrolysis as described by AOAC (2000) (42).

### Co-occurrence network analysis

Based on Spearman's correlation matrices, correlation networks were analyzed between the relative abundance of bacterial abundance at the genus level and the content of SCFAs, amino acids, fat, crush ash, and moisture at different gut locations in pigs. An analysis of network structures was performed using Gephi v0.9.2 software (43).

### Data analysis

We used GraphPad Prism 8 software to perform one-way analysis of variance (ANOVA). Data were expressed as means ± standard deviations (SD), and a *P* value < 0.05 was set as the level of statistical significance.

## Results

### Bacterial diversity

A total of 1,492,547 high-quality reads were generated from the 36 samples and were classified into 16,191 bacterial OTUs. Alpha diversity was observed in different gut locations. The samples from the large intestine (cecum and colon) had significantly higher Chao and Shannon indices than samples from the small intestine (jejunum and ileum) (Table 2, *P* < 0.05), suggesting that the richness and diversity of intestinal microbiota communities in pigs gradually increased from the small intestine to the large intestine in the digestive tract.

**Table 2:** Diversity of sequencing data of pig intestinal contents from different intestinal segments.

**Sample ID**	**Reads**	**OTU**	**Chao**	**Shannon**	**Simpson**
Jejunum	38 050 ± 2 228	420 ± 100^bc^	420 ± 100.31^bc^	3.88 ± 0.429^b^	0.08 ± 0.021 2^b^
Ileum	42 087 ± 3 873	243 ± 35^c^	243 ± 35.31^c^	2.75 ± 0.276^c^	0.22 ± 0.071 7^a^
Cecum	39 370 ± 2 771	434 ± 35^ab^	434 ± 35.22^ab^	4.41 ± 0.229^ab^	0.05 ± 0.015 1^b^
Ascending colon	46 195 ± 2 939	607 ± 30^a^	607 ± 29.96^a^	4.91 ± 0.158^a^	0.03 ± 0.006 9^b^
Transverse colon	41 266 ± 4 233	501 ± 38^ab^	501 ± 37.70^ab^	4.62 ± 0.146^ab^	0.04 ± 0.009 1^b^
Descending colon	41 790 ± 3 545	495 ± 39^ab^	495 ± 38.53^ab^	4.57 ± 0.189^ab^	0.04 ± 0.010 7^b^

All data are presented as the mean ± standard deviation (*SD, n* = 6). The same letters in each column indicate no significant difference (*p* > 0.05).

**Figure 1:**
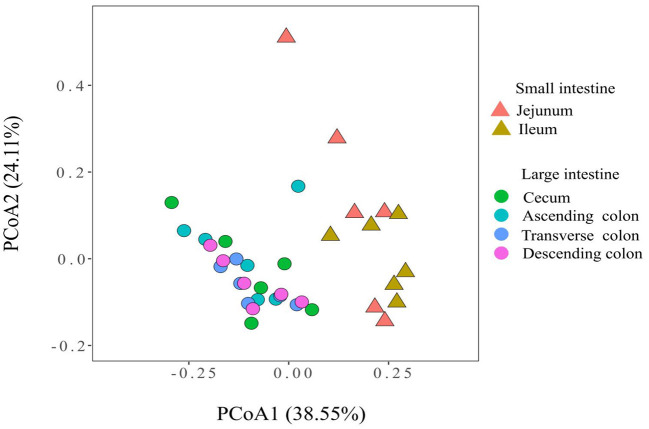
Principal coordinate analysis of the microbial communities based on the weighted UniFrac distance.

To measure the similarity between microbiota communities, a principal coordinate analysis (PCoA) was performed and revealed distinct clustering of the microbiota compositions of the different gut locations in pig (Figure 1). The community structures observed in the large intestine were significantly different from those detected in the small intestine.

### Microbial community composition in different intestinal locations

**Figure 2:**
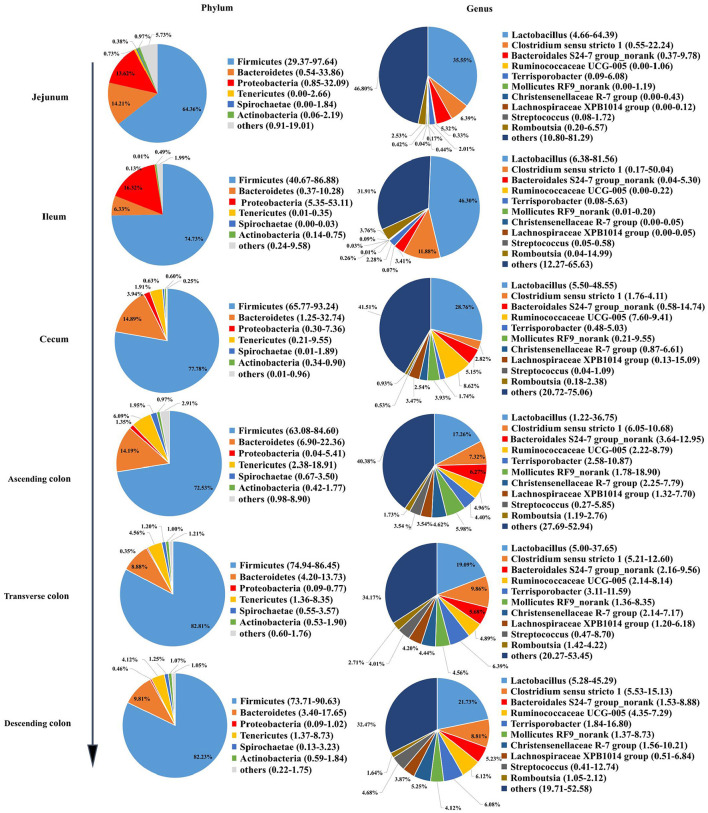
Microbial community structure in different intestinal locations of pigs at the phylum and genus levels.

To reveal the microbial composition in different intestinal locations, we calculated the abundances of the top 6 phyla and the top 10 genera in all pig samples. The dominant phyla were Firmicutes, Bacteroidetes, and Proteobacteria. Firmicutes was the most dominant phylum in different intestinal segments, representing 64.36 to 82.81% of the total microbial population in pigs (Figure 2). However, the abundance of Firmicutes (64.36%) in the jejunum was lower than that in other intestinal segments of pigs (> 70%). Firmicutes and Tenericutes were predominant in the cecum and colon of pigs and made up a smaller percentage in the jejunum and ileum. Otherwise, the abundance of Proteobacteria in the small intestine was significantly higher, ranging from 13.62 to 16.32% in the jejunum and ileum. At the genus level, the most abundant genera appeared to be very diverse in different guts. *Lactobacillus, Clostridium sensu stricto1, Bacteroidales S24-7* group norank, and *Ruminococcaceae UCG-005* were the dominant genera. *Lactobacillus* was present at higher levels in the small intestine than in the large intestine, and *Ruminococcaceae UCG-005* and *Mollicutes RF9_norank* were more abundant in the large intestine. The ileum had a higher abundance of *Lactobacillus* (46.30%) than the jejunum, cecum and colon.

**Figure 3:**
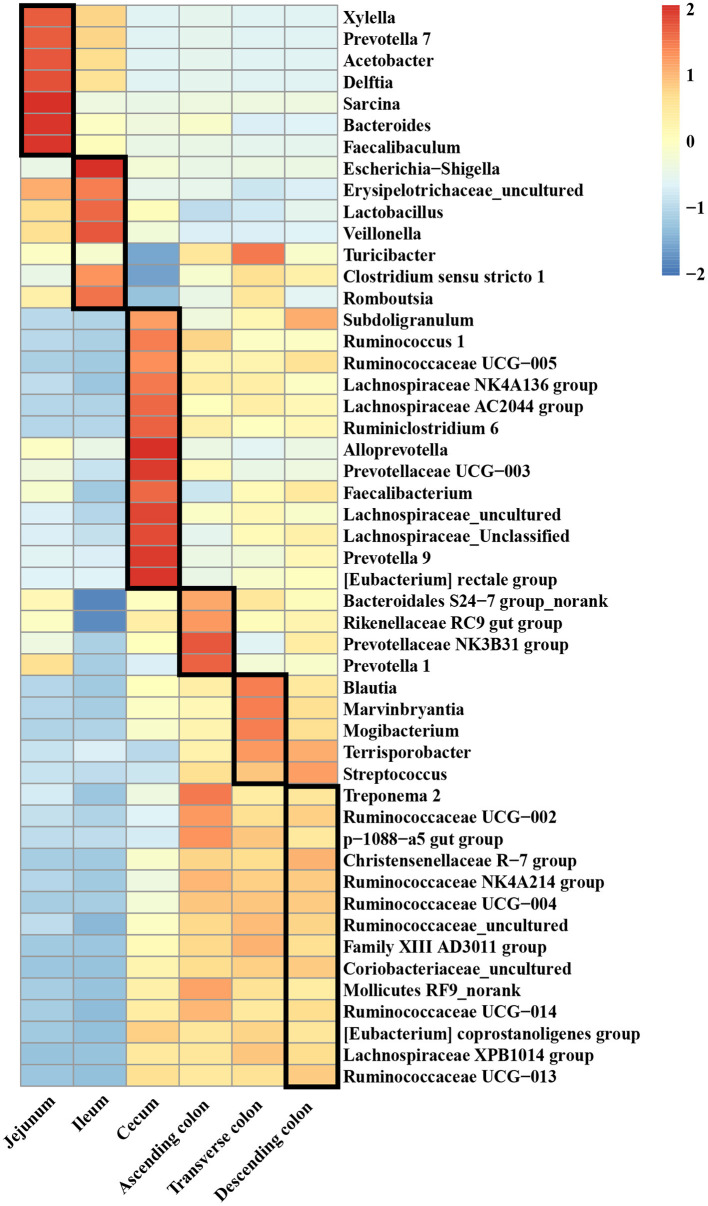
The top 50 genera in different intestinal locations of pigs.

A clustered heatmap based on the 50 most dominant genera is shown in Figure 3. The most abundant genera in the jejunum were *Sarcina, Bacteroides*, and *Faecalibacterium*, and the proportions of *Escherichia-Shigella, Lactobacillus*, and *Veillonella* were notably increased in the ileum. *Alloprevotella* and *Prevotellaceae* were the predominant genera in the cecum. For the different colon segments, the genera *Prevotellaceae NK3B31* group and *Prevotella 1* were considerably more abundant in the ascending colon; *Blautia, Marvinbryantia*, and *Mogibacterium* were enriched in the transverse colon; and *Christensenellaceae R-7* group, *Coriobacteriaceae*_*uncultured*, and *Ruminococcaceae UCG-013* had a relatively high relative abundance of bacteria in the descending colon.

**Figure 4:**
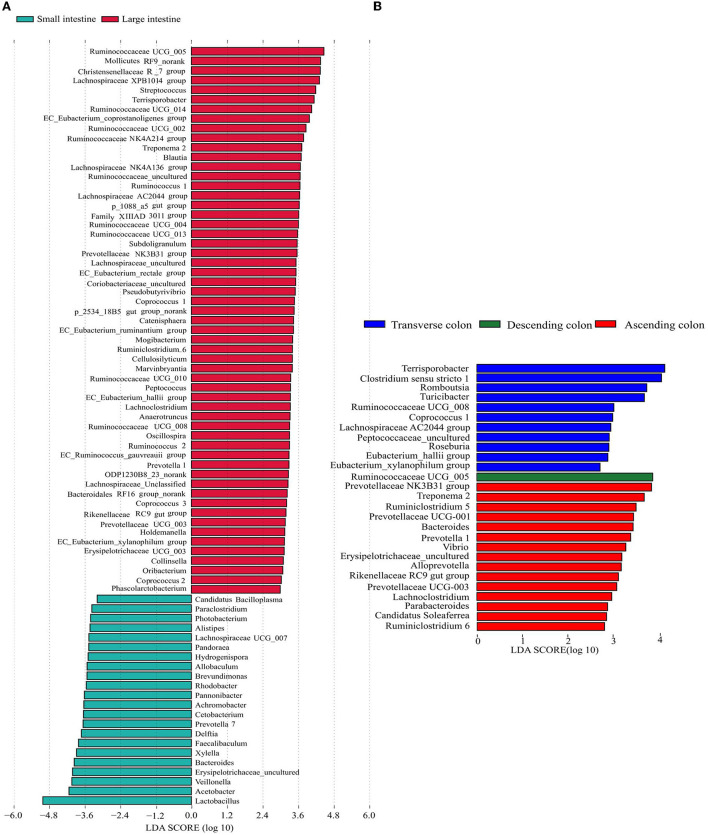
Differentially represented genera in different intestinal locations of pigs identified by LEFSe using an LDA score threshold of > 2.5. **(A)** plots the discriminative genera enriched in the small and large intestine, **(B)** plots the discriminative genera enriched in different colon segments. The vertical axis represents the names of different genera, and the colors correspond to different intestinal locations.

To further explore the specificity of the distribution of microflora in each intestinal segment, LEfSe analysis was used to identify the genera with a differential abundance between the small and large intestine, and then the analysis identified differentially abundant genera in different colon segments. In the large intestine, a total of 57 genera were significantly enriched, while only 22 genera were enriched in the small intestine (Figure 4A). The relative abundances of *Acetobacter, Veillonella, Bacteroides, Erysipelotrichaceae_uncultured, Xylella* and *Faecalibaculum* were more abundant in the small intestine, while the abundances of *Ruminococcaceae UCG-005, Mollicutes RF9*_norank, *Christensenellaceae R-7* group, *Lachnospiraceae XPB1014* group, *Streptococcus, Terrisporobacter* and *Ruminococcaceae UCG-014* were significantly higher in the large intestine. Concerning the different colon segments, the descending colon had higher proportions of *Ruminococcaceae UCG-005*, whereas *Terrisporobacter, Clostridium sensu stricto 1, Romboutsia, Turicibacter, Ruminococcaceae UCG-008, Coprococcus 1*, and *Lachnospiraceae AC2044* group were enriched in the transverse colon. In addition, *Prevotellaceae NK3B31* group, *Treponema 2, Ruminiclostridium 5, Prevotellaceae UCG-001, Bacteroides*, and *Prevotella 1* were more abundant in the ascending colon (Figure 4B).

### SCFAs in different intestinal locations and their correlation with the microbial community

**Figure 5:**
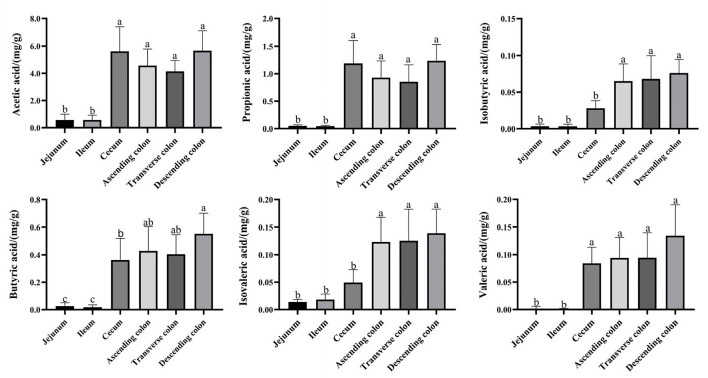
Concentrations of SCFA in different intestinal locations of pigs. Data are shown as mean ± *SD* (*n* = 6). The same letter within each column indicates no significant difference (*p* > 0.05).

**Figure 6:**
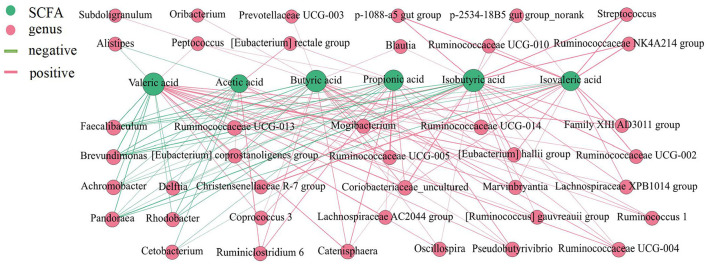
Correlation analysis between the microbial community and the concentrations of SCFAs in the intestine of pigs. The association between the top 100 genera and SCFAs were analyzed using Spearman's correlation method. The node size is proportional to the number of connections.

The total levels of SCFAs were significantly higher in the cecum and colon (Figure 5) (*P* < 0.05), and acetic acid, propionic acid, and butyric acid were the main metabolic products of the microbiota. To better understand the interaction between the microbiota and SCFAs, Spearman's correlation analysis was used to assess the association between the top 100 genera and SCFAs in different intestinal segments (Figure 6). The genera *[Eubacterium] hallii* group, *Mogibacterium, Ruminococcaceae UCG-005, Ruminococcaceae UCG-004, Pseudobutyrivibrio, Marvinbryantia, Catenisphaera, [Eubacterium] coprostanoligenes* group, and *Lachnospiraceae AC2044* group were positively correlated with a variety of SCFAs. The genera *Ruminococcaceae UCG-014, Ruminococcaceae UCG-013* and *Coriobacteriaceae_uncultured* also had a positive correlation with valeric acid, isovaleric acid, isobutyric acid, butyric acid, and propionic acid. Furthermore, *Brevundimonas and Pandoraea* showed a negative correlation with the contents of all kinds of SCFAs.

### Nutrients in different intestinal locations and their correlation with the microbial community

**Figure 7:**
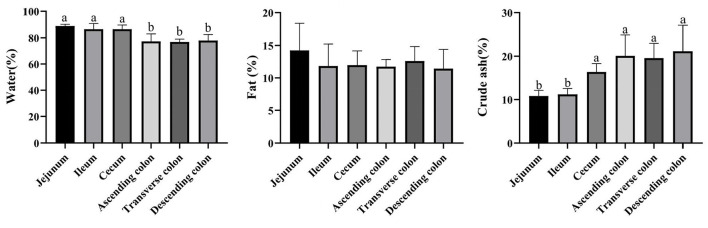
Concentrations of moisture, fat, and crude ash in different intestinal locations of pigs. The concentration of fat and crude ash was based on the dry matter of the intestinal contents. Data are shown as mean ± *SD* (*n* = 6). The same letter within each column indicates no significant difference (*p* > 0.05).

**Figure 8:**
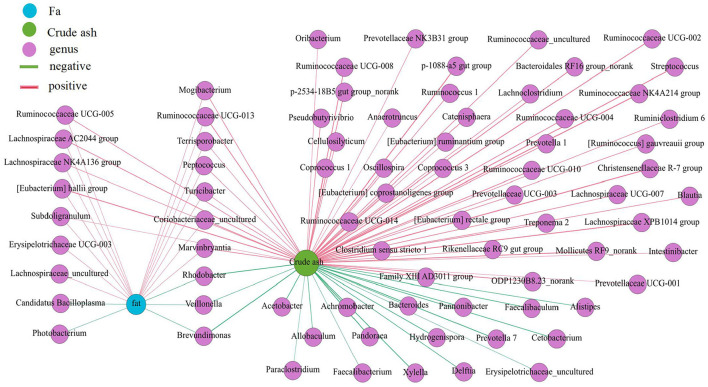
Correlation analysis between the microbial community and the concentration of fat and crude ash in intestine of pigs. The association between the top 100 genera and the fat and crude ash were analyzed using Spearman's correlation method. The node size is proportional to the number of connections.

The proportion of crude ash in the large intestine was significantly greater than that in the small intestine, and the colon had the highest content of crude ash (Figure 7) (*P* < 0.05). Additionally, the colon had a lower moisture content and approximately the same fat content in each intestinal segment. Next, we performed a network correlation (Figure 8) to analyze the association of the top 100 genera with nutrients in different intestinal segments of pigs. The genera *[Eubacterium] hallii* group, *Lachnospiraceae AC2044* group, *Ruminococcaceae UCG-005, Coriobacteriaceae_uncultured, Mogibacterium, Ruminococcaceae UCG-013, Terrisporobacter, Lachnospiraceae NK4A136* group, *Peptococcus, Turicibacter, Subdoligranulum and Marvinbryantia* showed a positive correlation with the contents of fat and crude ash, whereas the genera *Brevundimonas, Veillonella*, and *Rhodobacter* had a negative correlation.

**Figure 9:**
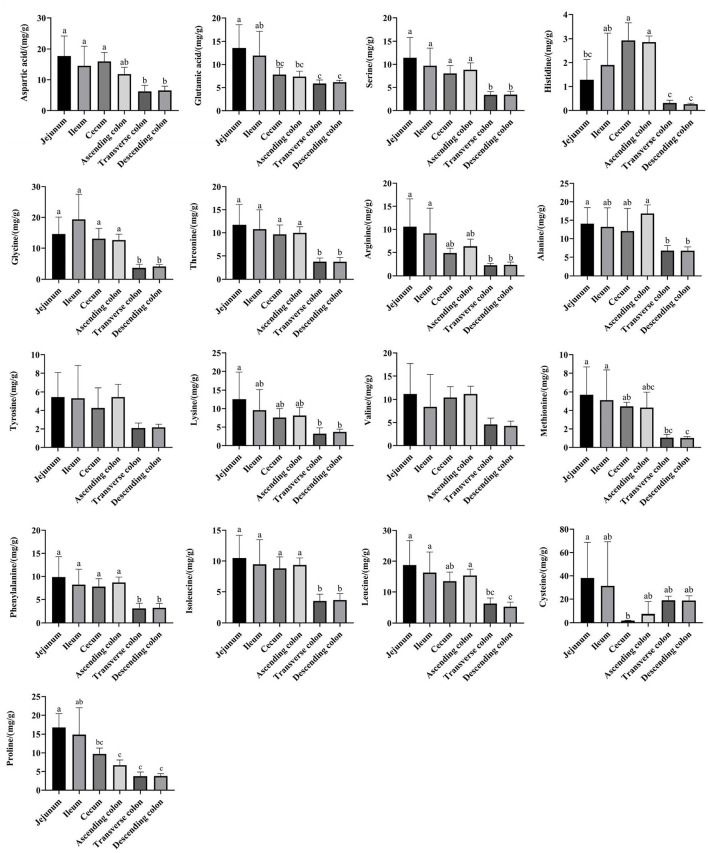
The concentrations of amino acids in different intestinal locations of pigs. The concentration was based on the dry matter of the intestinal contents. Data are shown as mean ± *SD* (*n* = 6). The same letter within each column indicates no significant difference (*p* > 0.05).

**Figure 10:**
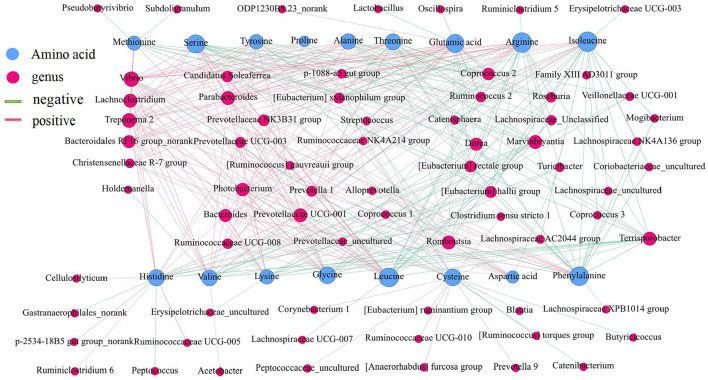
Correlation analysis between the microbial community and the concentrations of amino acids in the intestine of pigs. The association between the top 100 genera in the colon and the amino acids was analyzed using Spearman's correlation method. The node size is proportional to the number of connections.

The amino acid content in the small intestine was higher than that in the large intestine, while the amino acid content in the ascending colon was significantly higher than that in the transverse colon and descending colon (Figure 9) (*P* < 0.05). Then, a network correlation (Figure 10) was used to analyze the association of the top 100 genera with amino acids in the colon. The genera *Dorea, Marvinbryantia, Terrisporobacter, Romboutsia, [Eubacterium] rectale* group, and *[Eubacterium] hallii* group showed a negative correlation with the content of a variety of amino acids, indicating a robust contribution to the synthesis of amino acids. The genus *Ruminococcaceae UCG-005* was negatively correlated with histidine, *Lachnospiraceae AC2044* group, *Coriobacteriaceae_uncultured* and *Mogibacterium* and had a negative correlation with isoleucine and phenylalanine. The genera *Lachnoclostridium, Vibrio, Parabacteroides*, and *Treponema 2* showed a positive correlation with the contents of various amino acids.

**Figure 11:**
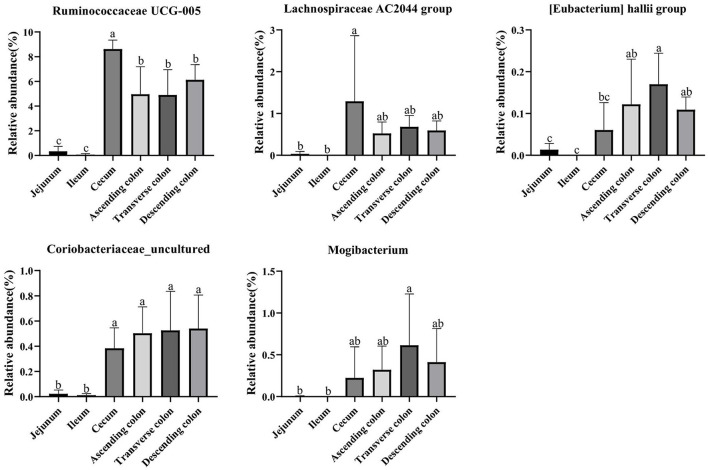
The relative abundance of *Ruminococcaceae UCG-005, [Eubacterium] hallii* group, *Lachnospiraceae AC2044* group, *Coriobacteriaceae_uncultured* and *Mogibacterium* in different intestinal locations of pigs. Data are shown as mean ± *SD* (*n* = 6). The same letter within each column indicates no significant difference (*p* > 0.05).

The genera *Ruminococcaceae UCG-005, [Eubacterium] hallii* group, *Lachnospiraceae AC2044* group, *Coriobacteriaceae_uncultured* and *Mogibacterium* clearly showed a positive correlation with the contents of SCFA, fat, and crude ash and a negative correlation with amino acids. Then, we analyzed the relative abundance of these genera in different intestinal location*s*. The results showed that *Ruminococcaceae UCG-005, [Eubacterium] hallii* group, *Lachnospiraceae AC2044* group, *Coriobacteriaceae_uncultured* and *Mogibacterium* were more abundant in the colon and cecum than in the jejunum and ileum (Figure 11) (*P* < 0.05).

## Discussion

The intestinal tract of humans and mammals is colonized by a dense and highly complex microbial community composed mainly of bacteria, which form a relatively dynamic and stable microecosystem in the intestinal environment and play an important role in the growth and health of the host (44–46). The intestinal microbiota is involved in nutrient absorption, metabolism and storage (47). Previous studies have shown that the intestinal microbiota contribute a lot in regulating the nutrient metabolism of hosts and maintaining energy balance (28, 48, 49). Due to the similarity between the human and pig microbiota and the knowledge that approximately 96% similarity of the functional pathways between the human and pig gut microbiota (34, 50), in this study, we used the pig as a model to analyze the microbial composition and contents of SCFA, amino acids, fat, and crude ash in different intestinal locations to explore the correlation between the intestinal microbiota and the metabolism and absorption of nutrients.

The diversity and abundance of the microbiota were higher in the large intestine than in the small intestine; simultaneously, the microbiota in the colon was the richest and the most diverse, which was consistent with the results of a previous study showing that the richness and diversity of the microbiota gradually increased along the digestive tract from the duodenum to the colon (51). PCoA showed that the microbiota in the small intestine clearly differed from that in the large intestine, which might be closely related to the physiological functions of each intestinal segment. Additionally, each region harbor specific microbial community (52). Proteobacteria were predominant in the jejunum and ileum and made up a smaller percentage in the cecum and colon, whereas the proportion of Firmicutes was higher than in the cecum and colon, which was consistent with the findings of several previous studies on the distribution of different intestinal microbiota in pigs (1) (53, 54). In contrast, the relative abundance of *Acetobacter, Veillonella, Bacteroides, Erysipelotrichaceae_uncultured, Xylella* and *Faecalibaculum* were higher in the small intestine of pigs, while *[Eubacterium], Ruminococcaceae, Mollicutes, Christensenellaceae, Lachnospiraceae, Streptococcus, Terrisporobacter* and *Ruminococcaceae* were enriched in the large intestine. The main reason for the unique microbiota in the difficult intestine is that from the proximal small intestine to the large intestine, a strong pH gradient and a large oxygen gradient can be observed (55, 56). The small intestine is a harsh microenvironment formicrobial life because of the shorter transit time and lower pH values and there is an increased proportion of aerobic or facultative anaerobic bacteria. In contrast, the large intestine dominantly hosts a number of anaerobic bacteria (57). Moreover, within the same intestinal segment, the distribution of bacteria in different spaces was also distinct. In this study, diversity of the microbial composition was found in different colonic locations, with the descending colon containing higher proportions of the *Ruminococcaceae UCG-005* while the transverse colon was enriched with *Terrisporobacter, Clostridium sensu stricto 1, Romboutsia* and *Turicibacter*, and the ascending colon had greater abundances of *Prevotellaceae NK3B31* group, *Treponema 2, Ruminiclostridium 5*. Numerous investigations have been conducted to examine the intestinal microbiota in different human intestinal segments and have shown that the jejunum is mainly composed of gram-positive aerobic bacteria, including *Lactobacillus, Streptococcus* and *Staphylococcus*; the number of ileal anaerobic bacteria exceeds aerobic bacteria; and more than 98% of the colon contains obligate anaerobic bacteria, mainly *Bacteroides, Bifidobacterium*, and *Eubacterium* (56, 58, 59). The similarity of bacterial structure leads to the similar nutrient absorption process between pigs and human sand the colon is the main place of bacterial fermentation between pigs and humans (35, 60), therefore the application of pigs in human nutrition model models is increasingly gaining traction.

SCFAs are considered indirect microbial metabolism substrates and involved in the regulation of energy metabolism, immunity, adipose tissue expansion and modulation of cancer cell development (61, 62). In our study, the concentrations of SCFA were higher in the large intestine than in the small intestine due to the slower rate of flow in the large intestine and metabolism favoring fermentation of indigestible carbohydrates; additionally, the anaerobic state of the colon provides an ideal environment for anaerobic digestion (56, 63). Previous studies have reported that anaerobic bacteria in the colon ferment undigested carbohydrates from the small intestine to produce SCFAs. Propionic acid is the main product of Bacteroidetes fermentation, and butyric acid is mainly produced by the metabolism of Firmicutes (64), in accordance with our findings. The genera *Ruminococcaceae* (*UCG-004, UCG-005, UCG-014, UCG-013*), *Coriobacteriaceae_uncultured, [Eubacterium]* (*hallii* group, *coprostanoligenes* group), *Mogibacterium, Pseudobutyrivibrio, Marvinbryantia, Catenisphaera*, and *Lachnospiraceae AC2044* group had a positive correlation with a variety of SCFAs based on the network analysis. Simultaneously, these genera were enriched in the large intestine, which suggested that the large intestine was the main region of SCFA production and that these genera were the core bacteria of fiber fermentation.

The crude ash and fat are important indicators to reflect nutrients. The content of crude ash is related to the absorption and utilization of minerals, the gut microbiota can affect the host mineral metabolism and participate in the metabolism of calcium, iron, magnesium, selenium, copper, and zinc (65–67). The fat content is involved in metabolism and body energy reserves and is an important index affecting human health (1, 68). Due to different digestive enzymes in the jejunum, ileum, cecum and colon, there are differences in digestion, absorption and utilization of nutrients in different intestinal locations (45, 56, 69). In our study, the proportion of crude ash in the large intestine was significantly greater than that in the small intestine. The genera *[Eubacterium] hallii* group, *Lachnospiraceae AC2044* group, *Ruminococcaceae UCG-005, Coriobacteriaceae_uncultured*, and *Mogibacterium* showed a positive correlation with fat and crude ash. The utilization of amino acids is widely distributed among bacteria residing in the digestive tract of humans and animals (18, 70), and the microbial community structure in different intestinal segments affects compartmentalized amino acid metabolism (54). In our study, the amino acid content was higher in the small intestine than the large intestine, while it was significantly higher in the ascending colon than in the transverse colon and descending colon, which is consistent with a previous study showing faster transit and a preference for amino acid metabolism in the small intestine, where the community primarily consisted of rapidly dividing facultative anaerobes such as Proteobacteria and Lactobacillales (71). In contrast, bacteria in the large intestine use amino acids mainly for catabolism. Bacteria in the large intestine of humans and animals can degrade a large number of amino acids, and the products of fermented amino acids, including short-chain and branched-chain fatty acids, can also participate in the energy metabolism of the host (72). The genera *Dorea, Marvinbryantia, Terrisporobacter, Romboutsia, [Eubacterium] rectale* group, *[Eubacterium] hallii* group, *Ruminococcaceae UCG-005, Lachnospiraceae AC2044* group, *Coriobacteriaceae_uncultured*, and *Mogibacterium* showed a negative correlation with the contents of a variety of amino acids in the colon. Moreover, most of these bacteria were enriched in the transverse colon and descending colon, making a robust contribution to the degradation of amino acids. The genera *Ruminococcaceae UCG-005, [Eubacterium] hallii* group, *Lachnospiraceae AC2044* group, *Coriobacteriaceae_uncultured* and *Mogibacterium* showed a positive correlation with the contents of SCFA, fat, and crude ash and a negative correlation with amino acids and these genera were mainly distributed in the colon and cecum.

## Conclusion

This work presents the first overview of the microbial community and nutrient metabolism in different intestinal locations using a pig model. The richness and diversity of intestinal microbial communities gradually increased from the small intestine to the large intestine. The concentrations of SCFA were higher in the large intestine than in the small intestine, and the concentrations of amino acids in the small intestine were higher than in the large intestine, while the amino acid content in the ascending colon was significantly higher than those in the transverse colon and descending colon. The *Ruminococcaceae UCG-005, Coriobacteriaceae_uncultured, [Eubacterium] hallii, Mogibacterium*, and *Lachnospiraceae AC2044* group had a positive correlation with the contents of SCFA, fat, and crude ash and a negative correlation with amino acids in different gut locations of pigs. Collectively, these correlation outcomes prompted us to further understand the relationship between the microbiota and nutrient metabolism in different gut environments, which is important for gut health and whole-body homeostasis.

## Accession number

The sequencedata of this study have been submitted to the NCBI Sequence Read Archive (SRA) database under the study accession number: PRJNA853391.

## Data availability statement

The datasets presented in this study can be found in online repositories. The names of the repository/repositories and accession number(s) can be found below: https://www.ncbi.nlm.nih.gov/, PRJNA853391.

## Ethics statement

The animal study was reviewed and approved by (ZAAS-2017-009).

## Author contributions

Experiment design: KC, LL, YX, and YS. Animal experiments: YS, XZ, and GZ. Data analysis and visualization: YS, XD, and YX. Roles, writing—original draft, writing—review and editing: YS and YX. All authors contributed to the article and approved the submitted the manuscript.

## Funding

This work was supported by the National Natural Science Foundation of China (31972999), Development Center of Animial Husbandry and Agricultural Machinery in Jinhua City (OBANG2021-FW030-ZFCG0321), State Key Laboratory for Managing Biotic and Chemical Threats to the Quality and Safety of Agro-products, Zhejiang Academy of Agricultural Sciences (2010DS700124-ZZ1905).

## Conflict of interest

The authors declare that the research was conducted in the absence of any commercial or financial relationships that could be construed as a potential conflict of interest.

## Publisher's note

All claims expressed in this article are solely those of the authors and do not necessarily represent those of their affiliated organizations, or those of the publisher, the editors and the reviewers. Any product that may be evaluated in this article, or claim that may be made by its manufacturer, is not guaranteed or endorsed by the publisher.
